# Genetic advances in Meniere Disease

**DOI:** 10.1007/s11033-022-08149-8

**Published:** 2022-12-24

**Authors:** Qingqing Dai, Lili Long, Hui Zhao, Ruikai Wang, Hong Zheng, Maoli Duan

**Affiliations:** 1 Department of Otorhinolaryngology-Head and Neck Surgery, West China Hospital of Sichuan University, Chengdu, 610041 Sichuan China; 2 Department of Otolaryngology-Head and Neck, Department of Clinical Science, Intervention and Technology, Karolinska University Hospital, Karolinska Institute, 17176 Stockholm, Sweden; 3 Department of Otorhinolaryngology, Sichuan University Hospital of Sichuan University, Chengdu, 610065 Sichuan China; 4 Department of Otorhinolaryngology, Hospital of Civil Aviation Flight University of China, Guanghan, 618300 Sichuan China; 5 West China School of Medicine, Sichuan University, Chengdu, 610041 Sichuan China

**Keywords:** Meniere Disease, Gene, Etiology, Pathogenesis

## Abstract

Meniere Disease (MD) is an idiopathic inner ear disease with complex etiology and pathogenesis, which is still unclear. With the development in gene analysis technology, the genetic research of MD has attracted extensive attention, resulting in a large number of studies on the research of the relationship between human genes and MD. This paper aims to review the studies on this topic in recent years. The studies mainly focused on the genetics of familial MD and the correlation between MD and potentially related functional genes. The results of these studies have demonstrated the complexity and diversity of the pathogenesis of MD with both genetic and epigenetic alterations, suggesting that MD might be related to inflammation, immunity, aqua and ion balance in the lymphatic fluid, virus infection, metabolism, and abnormal function of nerve conduction. The finding of rare mutations in *TECTA*, *MYO7A* and *OTOG* genes and other genes such as *CDH23*, *PCDH15* and *ADGRV1* in the same families suggest that the integrity of the stereocilia and their interaction with the tectorial and otolithic membranes could be involved in the pathophysiology of familial MD.

## Introduction

Meniere Disease (MD) is one of the most common disorders in otolaryngology, audiology and neurotology, first reported by Meniere Prosper in 1861, with endolymphatic hydrops as fundamental pathological basis. MD is characterized by intermittent paroxysm, fluctuating sensorineural hearing loss, tinnitus, and ear fullness, with unknown etiology and pathogenesis, and is associated with autoimmunity, mental stress, viral infection, anatomical factors, trauma, and genetic factors. One of the characteristics of MD, especially familial Meniere Disease (FMD) is the differences in incidence among races, regions, and family aggregation, suggesting that this disease may be related to genetic factors [1–7]. Many scholars believe that MD may be a multifactorial disease caused by interactions between one or more genes and environmental factors. Therefore, lots of researches have been done exploring genetics of MD, which mainly focused on FMD and the correlations between MD and functional genes. In this review paper, we focus on the progress in understanding the genetics of MD in details.

## Genetic studies of FMD

### Gene mapping analysis

Gabrikova et al. conducted a gene linkage analysis of a Swedish autosomal dominant FMD family and located the MD gene at 12p2.3 [8], but the results were inconsistent with those of a Finnish FMD linkage analysis [9]. The team used allele and haplotype association analysis to further locate MD to a 1.48 Mb region on 12p2.3 containing *RERGL* and *PIK3C2G* genes. *RERGL* gene is a member of the Ras family and is involved in cell signaling. As a member of the PI3Ks family, the *PIK3C2G* gene is involved in cell proliferation and other regulatory processes. The roles of the two genes in MD need to be further studied [10]. Arweiler et al. [11] located the MD gene on chromosome 5 based on linkage analysis results of 17 MD families. Fung et al. [12] located FMD-related genes in the HLA gene group of chromosome 6p through linkage analysis of two families. Frejo et al. [13] used immune genotyping array analysis to locate the MD target gene at 6p21.33, and the leading signal in the locus 6p21.33 was rs4947296. This region is a trans-expressed quantitative trait locus, possibly mediating the inflammatory response of MD by increasing NF-κB translation.

### FMD candidate genes

Lopez-Escamez and his team sequenced the whole exome of FMD patients in Spain, and reported five candidate genes in four autosomal dominant FMD families. They described variants in two candidate genes, *FAM136A* and *DTNA* in a single family [2], a missense variant in gene *PRKCB* in another family that segregated the hearing loss phenotype [3], and rare heterozygous variants in the genes *DPT* and *SEMA3D* in another 2 families [4]. In addition, multiple rare missense mutations of the *OTOG* gene were found in 33% of familial MD, suggesting multiple allelic inheritances [5]. Mutation of *FAM136A*, *DTNA*, and *DPT* genes also appeared in some sporadic Meniere Disease (SMD) patients in South Korea [14]. Lopez-Escamez and his team also observed multiple families carrying rare variants in genes encoding proteins involved in the structure of the hair cells stereocilia and their attachment to the tectorial membrane (TM): six FMD families carrying rare missense heterozygous variants or a short deletion in the coding region of the *TECTA* gene encoding protein α-tectorin, which is one of the main non-collagenous proteins of the TM [5]; mutations in the *MYO7A* gene encoding cilia-motor proteins in inner ear hair cells co-segregated with some novel and rare variants in other genes involved in the organization of the stereocilia links such as *CDH23*, PCDH15 or *ADGRV1* in nine Spanish and Swiss MD families [6]; 15 unrelated MD families carrying an enrichment of rare missense variants in the *OTOG* gene [5].

*COCH* gene, located at 14q12-13, encoding glycoprotein specific to acellular membranes of the inner ear and maybe involved in the organization and/or stabilization of the fibrillar network that compose the TM in the cochlea is an autosomal dominant gene for non-syndromic deafness. This gene mutation can cause the disease DFNA9 [15]. At least 10% of autosomal negative inherited non-syndromic sensorineural hearing loss is caused by DFNB16B gene mutation in *STRC* [16]. Both of them were accompanied by vestibular dysfunction and could show MD symptoms.

In addition, point mutations of *EGFLAM* and *ITGA8* genes in autosomal dominant FMD families in China, P.y273N and P.L229F mutations of *HMX2* genes in Finnish FMD families, and meaningless mutations of *LSAMP* genes in Iranian autosomal recessive FMD families have been reported [17–19].

FMD is inherited in an autosomal dominant, autosomal recessive, or mitochondrial genetic manner, and the differences in these findings suggest genetic heterogeneity among families of MD. These candidate genes are mostly expressed in nerve or inner ear tissues. Therefore, the pathogenesis of FMD may be to hinder nerve development, maintenance, or functional recovery by reducing the nutrient support of nerve endings to hair cells; influence neurite formation and growth; damage the inner ear cell structure; or interact with various proteins to affect hearing through changing lysosomal dynamics.

## Study on the relationship between MD and genes

### Immunity-, inflammation-, and stress-related protein genes

#### MD and histocompatibility leukocyte antigen genes

Major histocompatibility complex (MHC) is a group of genes encoding major histocompatibility antigens in animals. Human MHC is called histocompatibility leukocyte antigen (HLA). HLA gene complex located at 6p21.31 has a complex genetic polymorphism and is closely related to the immune response. The gene group can be divided into three types: Class I genes include HLA-A, B, C, E, and F, which exist on the surface of nucleated cells; Class II gene HLA-D consists of HLA-DR, DQ, and DP subregions, which mainly located on the surface of antigen-presenting cells; Class III genes are mostly complement-related genes, tumor necrosis factor (TNF), heat shock proteins (HSP), and transcription factor genes, which are located between class I and II genes. HLA-II genes are known to play important roles in most autoimmune diseases. Major Histocompatibility Complex Class I chain-related gene A (*MICA*) genes are atypical Class I MHC genes. MICA binds to receptors of some immune cells as a ligand, participating in immune response, and may playing an essential role in autoimmune diseases. Immunogical factors are considered to play an important role in the pathogenesis of MD, so there are many studies on the correlation between HLA and MD [20–29] (Table 1).

**Table 1:** Studies on the correlation between MD and histocompatibility leukocyte antigen (HLA) genes

Object	Gene	Result	Source
Spanish	*HLA*	Not correlated with MD	[22]
	MICA*A.4	Protective for MD	[27]
	HLA-DRB1*1101	Not correlated with BMD	[26]
Mediterranean, Spanish	*HLA-DQB1*	Not correlated with BMD	[26]
Mediterranean	HLA-DRB1*1101	Susceptible for BMD	[26]
Caucasian	HLA-Cw*04	Susceptible for MD	[20]
	HLA-Cw*07	Susceptible for MD	[21]
Asian	HLA-DRB1*1602	Susceptible for MD	[22]
	HLA-Cw*04	Susceptible for MD	
Chinese	HLA-DRB1*09	Protective for MD	[29]
	HLA-A*11	Susceptible for MD	[28]
South Korean	HLA-Cw*0303	Susceptible for MD	[23]
	HLA-DRB1*15	Susceptible for MD	
	*HLA-B44*	Protective for MD	
	HLA-DRB1*0405	Susceptible for MD	[24]
	HLA-DRB1*1201	Susceptible for MD	
	HLA-DRB1*13	Protective for MD	
American	*HLA-B27*	Susceptible for BMD	[25]
	*HLA-DR2*	Protective for MD	[22]
British	HLA-Cw*07	Susceptible for MD	[22]

HLA association studies have not been replicated and they are pending confirmation. The differences in HLA expression among ethnic groups have not been ruled out. Furthermore, there are few large sample studies. Therefore, no significant breakthrough has been made. Whether HLA gene mutation is one of the causes of MD needs to be confirmed.

### Other immunity-, inflammation-, and stress-related protein genes

In addition to HLA, a large number of other genes related to immunity, inflammation, and oxidative stress have been studied on the relationship with MD by scholars [7, 30–40] (Table 2). Lopez-Escamez et al. [31] found that protein tyrosine phosphatase non-receptor type 22 (*PTPN22*) gene 1858 C/T, which strongly negatively affects T cell activation may be responsible for BMD among the Hispanic population. This finding supports the hypothesis that autoimmunity is one of the etiological factors of BMD. It was found that interleukin-1 A (*IL-1 A*) gene polymorphism, *MIF*-173 G/C gene polymorphism, and *HSPA1A* gene polymorphism were associated with MD susceptibility in the Japanese population [32, 38, 41]. In addition, Toll-like receptor 10 (*TLR10*) is involved in non-specific immune responses and the variant rs11096955 of *TLR10* gene was associated with a better hearing prognosis in Spanish and Italian patients. NF-κB pathway gene *NFKB1*, histamine H4 receptor gene (*HRH4*), mediating the regulation of pro-inflammatory factors, and inflammatory disease-related gene *RANTES* were also found to be associated with MD [7, 35–37]. However, no studies have replicated these results.

**Table 2:** Studies on the correlations between MD and other genes related to immune, inflammatory, and oxidative stress proteins

Object	Gene	Function	Loci	Result	Source
Spanish	*TNF-α, MIF, INF-γ*	Pro-inflammatory factors	MIF (rs35688089), IFN-γ (rs2234688), TNF-α (rs1800629)	Not correlated with MD	[33]
	*NFKB1*	NF-κB pathway associated with inflammatory regulation, innate immunity, and adaptive immunity	rs3774937, rs4648011	Associated with the progression of hearing loss in unilateral MD	[7]
	*PTPN22*	Encodes lympho-specific tyrosine phosphatase, with a strong negative regulatory effect on T-cell activation	1858 C/T	Susceptible for BMD	[31]
	*CTLA4*	Inhibition of T cell activation through interaction with ligands	49 A/G	Not correlated with BMD	
Mediterranean, Spanish	*CD16, CD32*	Transmembrane glycoprotein, a low-affinity Fc receptor associated with immunity	CD16A(rs396991), CD32A(rs1801274)	Not correlated with MD	[34]
Spanish,Italian	*TLR10*	A class of important protein molecules involved in nonspecific immunity	rs11096955	Protective for MD	[35]
American, Mediterranean	*NOS1, NOS2A*	Oxidative stress, mediates the loss of spiral neurons	NOS(rs41279104, rs2682826 and cytosine adenosine microsatellite repeats in exon 1f) NOS2A (rs3833912)	Not correlated with MD	[39]
Japanese	*IL1A*	Play an important role in inflammatory response, transmit information, activate and regulate immune cells, mediate activation, proliferation, and differentiation of T and B cells	– 889 C/T; rs1800587	Susceptible for MD	[32]
	*IL1B*		– 511 C/T; rs16944	Not correlated with MD	
	*MIF*	Pro-inflammatory factor gene	– 173 G/C	Susceptible for MD	[43]
	*HSPA1A*	Intracellular protective proteins expressed by the body in response to stresses	190 G/C	Susceptible for MD	[38]
	*GPX1, PON1, PON2, SOD2*	Antioxidant enzymes In vivo; peroxidase decomposition enzyme; paraoxonase and superoxide dismutase	GPX1)(rs1050450), PON1(rs662, rs854560), PON2 (rs7493), SOD2(rs4880)	Not correlated with MD	[40]
Chinese	*HRH4*	Highly expressed in the immune system, mediates the regulation of pro-inflammatory factors	rs77485247	Associated with vertigo in MD and pro-inflammatory factor levels in blood	[36]
Iranian	*RANTES*	Associated with inflammatory disease	– 403 A	Protective for male MD	[37]
	*TNF-α*	Pro-inflammatory factor gene	– 238 A/G	Susceptible for MD	[30]

### Aquaporin and ion channel protein-related genes

Aquaporin subtypes 1–5, 7, and 9 are expressed in inner ear tissues to transport water and small soluble molecules such as glycerol. Potassium channel protein KCNE plays an important role in transmembrane ion and water transport in the inner ear. The pathological basis of MD is endolymphatic hydrops, suggesting that aquaporin and ion channel protein-related genes may be associated with MD. However, studies on *AQP* and *KCNE* gene polymorphisms showed opposite results in different populations (Table 3) [1, 42–49]. A meta-analysis based on current published studies showed that the *KCNE1* rs1805127 and *KCNE3* rs2270676 variants are not associated with the risk of MD [49]. Further replication studies in distinct populations are required to confirm the ethnic stratification of the association. Whether these two genes are related to MD needs to be confirmed by further large-sample studies.

Besides the genes mentioned above, Na+, K+-ATPase and sodium-calcium exchanger are vital to maintaining the ion balance of endolymph. In addition, Adducin is a kind of cytoskeleton protein, which contain subunits α, β, and γ, encoded by genes *ADD1*, *ADD2*, and *ADD3*, respectively. We already know that the gene polymorphism of *ADD1* is related to salt-sensitive hypertension. Furthermore, the *SIK1* gene encodes salt-induced kinase 1, which is related to Na+, K+-ATPase. Moreover, the *SLC8A1* gene encodes a sodium-calcium exchanger. Teggi et al. found that *ADD1*, *SIK1*, and *SLC8A1* gene polymorphisms were associated with MD. These results support the hypothesis that ion transport decompensation may cause MD (Table 3) [50, 51].

**Table 3:** Studies on the correlations between MD and aquaporin, ion channel protein-related genes

Classification	Gene	Loci	Object	Result	Source
Aquaporin genes	*AQP2*	rs426496	Brazilian	Correlated with MD	[42]
	*AQP3*	rs591810	Brazilian	Correlated with MD	[42]
		Homozygous c.105G->C	Swiss	Maybe correlated with MD	[44]
	*AQP4*	rs2075575	Japanese	Not correlated with MD	[45]
	*AQP5*	– 1364 A/C	Caucasian	Not correlated with MD	[43]
		The variant G allele of rs3736309	Japanese	Correlated with MD	[45]
Potassium channel genes	*KCNE1*	rs1805127, rs1805128, rs17173510	Brazilian	Correlated with MD	[42]
		112G/A	Japanese	Correlated with MD	[46]
			Caucasian	Not correlated with MD	[1]
		rs1805127	Finnish	Correlated with SMD, not correlated with FMD	[47]
		653 C/T	Chinese	Correlated with SMD	[48]
	*KCNE3*	198T/C	Japanese	Correlated with MD	[46]
			Caucasian	Not correlated with MD	[1]
		492 A/C	Chinese	Correlated with FMD	[48]
Other ion transport associated genes	*ADD1*	rs4961	Italian	Correlated with MD	[50]
	*ADD2*	rs4984		Not correlated with MD	
	*ADD3*	rs3731566		Not correlated with MD	
	*SIK1*	rs3746951	Caucasian	Correlated with MD	[51]
	*SLC8A1*	rs487119			

### Virus-associated genes

Host cytokine C1 (HCFC1) interacts with herpes simplex virus proteins and is involved in viral replication in nerve cells. Vrabec et al. [52] found that the frequency of the primary allele of *HCFC1* SNPs increased in MD patients, and the secondary allele was a protective gene, suggesting that herpes virus infection might be a potential cause of MD.

### Other candidate genes

Lopez-Escamez’s team studied hundreds of patients with SMD in Spain and found that they had a high concentration of sensorineural deafness mutations in genes including *GJB2*, *USH1G*, *SLC26A4*, *ESRRB* and *CLDN14*. In addition, a rare synonymous mutation was found in another nonsyndromic deafness-related gene *MARVELD2* among several unrelated MD patients, whose role in MD was unknown [53]. Missense mutations of axon-oriented signaling pathway-related genes *NTN4* and *NOX3* were also found [54]. We found low frequency of *PARP-1* long allele (CA) < SUB > 17–20</sub > in BMD patients, suggesting its protective role in BMD [55].

Genetic variations of mitochondrial ribosomal genes *TFB1M* and *MRPS12* are not associated with hearing loss in MD [56]. Polymorphisms of *MTHFR* C677T and A1298C genes associated with folic acid metabolism are related to susceptibility for MD in the Japanese population [57]. Polymorphism of Caveolin 1 (*CAV1*) gene, which encodes protein interacting with estrogen, was found to be significantly correlated with MD, suggesting that estrogen may be associated with the pathogenesis of MD [58].

In conclusion, SMD genetic variations are more complex than FMD. The probably related genes are inflammation-, autoimmune-related genes, ion channel-related genes, virus related genes, and nerve, metabolism-related genes, prompt that its pathogenesis may be related to inflammation, immunity, virus infection, both inside and outside aqua and ion balance in the endolymph, metabolism, and abnormal function of nerve conduction.

### Epigenetic studies of MD

Shew et al. [59] studied the miRNA in lymphatic fluid and serum of 10 patients with MD. They found that the levels of aquaporin, and inflammation & autoimmunity pathway-related proteins were higher than those in the control group, suggesting that the pathogenesis of MD may be related to both water imbalance and dysfunction of the immune system.

Flook et al. [60] detected many differentially expressed methylated CpG islands in blood monocytes of Spanish SMD patients, some of which existed in causative genes of hearing loss, such as *PCDH15*, *ADGRV1*, and *CDH23*, while CpG island methylation deficiency existed in *PHB* genes. Combined with bioinformatic analysis, These epigenetic changes are considered to be associated with abnormal nerve electrical activity and inflammation in the inner ear.

## Conclusion

The results of genetic studies on MD have demonstrated the complexity and diversity of the pathogenesis of MD, suggesting that MD might be related to inflammation, immunity, aqua and ion balance in the lymphatic fluid, virus infection, metabolism, and abnormal function of nerve conduction. The genetic study of MD is still facing various difficulties and challenges. Due to the low incidence of MD in many countries and races, the main subject of genetic research is the Caucasian population with a high incidence of MD. The period of collecting FMD patient samples is quite long due to the middle-aged onset of MD. In addition, due to the limitation of medical conditions and levels, so as the lack of understanding of MD, it is difficult to diagnose MD in many hospitals, hinding MD sample collection. Furthermore, few epigenetic studies have been performed on MD.

In conclusion, in the future, more races, more large samples of the disease, and more special testing techniques are needed to study the target genes at both genetic and epigenetic levels, in order to achieve more extensive and in-depth research.
